# Longitudinal Study of the Relationship of Emotional Intelligence, Burnout, and Wellness of Emergency Medicine Residents in a Community Hospital.

**DOI:** 10.51894/001c.143945

**Published:** 2025-09-30

**Authors:** Christopher Kocharians

**Affiliations:** 1 Henry Ford Genesys

## 26

### INTRODUCTION

Resident burnout and wellbeing remain a focus of high priority and interest, in particular for emergency department (ED) residents. We conducted a 7 year longitudinal study of emotional intelligence (EI), Maslach Burnout Inventory (MBI), and physical well-being Inventory (PWI) of ED residents to identify the relationships between the EI and the MBI/PWI over the years of training.

### METHDOS

This prospective longitudinal study between 2017-2024 of 29 ED residents entering a four-year ACGME accredited program at a community hospital. The surveys (EI, MBI, and PWI) were given four times during the first year and once during the subsequent years. Consent was obtained from all residents before collecting the data. Institutional review board approval was obtained.

### RESULTS

Twenty-nine ED residents were voluntarily consented to the surveys: EI survey across time and gender showed an improvement from 4.36 (±0.36) to 4.97 (±1.03). The difference in male and female responses to the surveys were not statistically significant.

MBI constructed, emotional exhaustion (EE) changed from 14.15 (±7.79) at T1 to 23.75 (± 12.75) at T4 and TF at 22.97 (±13.82) (P=0.86). Depersonalization (DP) changed from 7.19 (± 4.73) to 10.00 (±6.24) to 9.52 (±0.69) (P=0.39) and Personal Accomplishment (PA) changed from 40.00 (±5.99) to 36.13 (±9.98) and finally get to 36.00 (P=0.01).

PWI Construct, career purpose (CP) changed from 21.36 (± 2.56) to 20.17 (± 3.93), and finally to 20.00 (±3.87) (p=0.58), distress (D) changed from 12.86 (±3.36) to 15.39 (±4.39) and final at 14.38 (±5.07) (P=0.24), and cognitive flexibility (CF) changed from 17.46 (±1.73) to 17.30 (±1.54) to finally at 16.97 (± 2.39) (P=0.33). Also changed in the same direction as MBI.

### CONCLUSION

Residents at PGY-1 level exhibited significant changes in burnout at the end of the year, which improved slightly in subsequent years. Support during the first years of training is highly recommended.

